# Molecular evolution of the enzymes involved in the sphingolipid metabolism of *Leishmania*: selection pressure in relation to functional divergence and conservation

**DOI:** 10.1186/1471-2148-14-142

**Published:** 2014-06-21

**Authors:** Vineetha Mandlik, Sonali Shinde, Shailza Singh

**Affiliations:** 1 National Centre for Cell Science, NCCS Complex, Pune University Campus, Ganeshkhind, Pune 411007, India

**Keywords:** Evolutionary biology, Sphingolipid metabolism of *Leishmania*, Functional divergence and conservedness, Specificity determining positions, Selection pressure, Codon usage bias, Relative synonymous codon usage, Effective number of Codon, GC content

## Abstract

**Background:**

Selection pressure governs the relative mutability and the conservedness of a protein across the protein family. Biomolecules (DNA, RNA and proteins) continuously evolve under the effect of evolutionary pressure that arises as a consequence of the host parasite interaction. IPCS (Inositol phosphorylceramide synthase), SPL (Sphingosine-1-P lyase) and SPT (Serine palmitoyl transferase) represent three important enzymes involved in the sphingolipid metabolism of *Leishmania.* These enzymes are responsible for maintaining the viability and infectivity of the parasite and have been classified as druggable targets in the parasite metabolome.

**Results:**

The present work relates to the role of selection pressure deciding functional conservedness and divergence of the drug targets. IPCS and SPL protein families appear to diverge from the SPT family. The three protein families were largely under the influence of purifying selection and were moderately conserved baring two residues in the IPCS protein which were under the influence of positive selection. To further explore the selection pressure at the codon level, codon usage bias indices were calculated to analyze genes for their synonymous codon usage pattern. IPCS gene exhibited slightly lower codon bias as compared to SPL and SPT protein families.

****Conclusion**:**

Evolutionary tracing of the proposed drug targets has been done with a viewpoint that the amino-acids lining the drug binding pocket should have a lower evolvability. Sites under positive selection (HIS20 and CYS30 of IPCS) should be avoided during devising strategies for inhibitor design.

## Background

*Leishmania*, a protozoan parasite is responsible for causing the infectious disease Leishmaniasis. Around 12 million people are affected by this disease worldwide. The sphingolipid metabolism of vertebrates, fungi and plants has been well documented. Sphingolipid metabolism in parasites like *Leishmania* plays an important role in maintaining the infectivity of the parasite. Sphingolipids form an integral component of the parasitic membranes [1]. They are localized in the membrane micro domains and are involved in a wide array of signal transduction pathways [2] Parasites like *Leishmania* scavenge host sphingolipids and remodel them into parasite specific sphingolipids. The key enzymes involved in the sphingolipid metabolism are a) SPT (Serine pamitoyl transferase) b) SPL (Sphingosine 1-P phosphate) and c) IPCS (Inositol phosphorylceramide synthase) [3].

**Figure 1:**
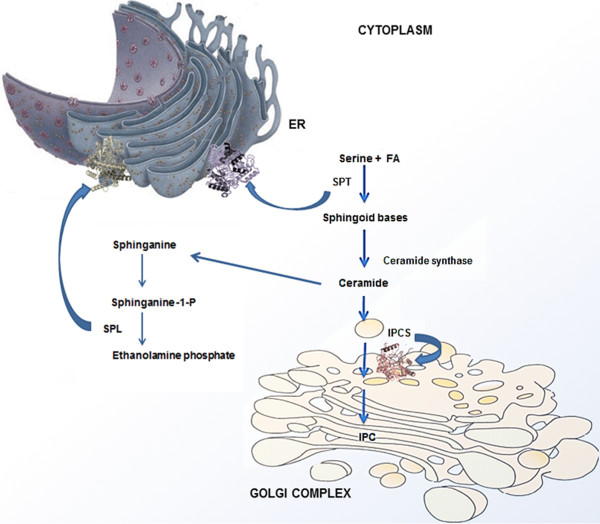
**Functional importance of the enzymes (IPCS, SPT and SPL) in the sphingolipid metabolism of *Leishmania*.**

SPT, the first key enzyme in the sphingolipid metabolism localizes into the endoplasmic reticulum and belongs to the class-II pyridoxal-phosphate-dependent aminotransferase family [4]. It comprises of two subunits SPTLC2 and SPTLC3 and catalyses the first step of the de novo synthesis of sphingolipids i.e. condensation reaction of serine and palmitoyl-CoA to form 3-dehydro D-sphinganine. The second key enzyme, SPL belongs to the Group II pyridoxal- dependent decarboxylase family of enzymes [5]. It acts on the phosphorylated sphingoid bases (PSBs) such as sphingosine-1-phosphate and cleaves them into aldehydes and phosphoethanolamine [6]. IPCS represents another key enzyme in the sphingolipid metabolism. IPCS is unique to certain fungi, plants and protozoan parasites like *Leishmania*. There is no mammalian equivalent of this enzyme and thereby IPCS has been considered as an attractive drug target in the sphingolipid metabolism of *Leishmania*[7]. IPCS localizes in the Golgi complex and catalyses the reaction involving the conversion of ceramide to inositol phosphoryl ceramide (IPC) [8,9]. The importance and role of these three target proteins in the sphingolipid metabolism has been shown in Figure 1.

The sphingolipid metabolism of *Leishmania* and many other pathogens is highly conserved and offers a series of attractive drug targets for further inhibitor design. IPCS, SPT and SPL in *Leishmania* have been identified as important target proteins by biochemical network modeling. [10] We present the phylogenetic relationship among these key enzymes in *Leishmania* to obtain a comparative history of the related proteins. Role of selection pressure, assessment of the strength of purifying versus diversifying selection for all the three target proteins has provided an idea of the molecular evolution of target proteins.

## Methods

### Acquisition of sequences

Amino acid and coding sequences of the three enzymes (IPCS, SPL and SPT) in the sphingolipid metabolism were retrieved from NCBI, ENSEMBLE and UNIPROT databases. A total of 54 sequences for IPCS and SMS, 40 sequences of SPL and 78 sequences for SPT were used in this study (Additional file 1: Table S1A-E).

### Sequence alignment, selective constraints and phylogenetic analysis

The sequences were aligned using CLUSTALW v2.0.9 using the default parameters. Larkin et al. [11] Phylogenetic tree reconstruction for the sphingolipid metabolism was done using MEGA 5 program by the Neighbour-Joining method with 10000 bootstrap resampling’s. Saitou and Nei [12,13]. The evolutionary distances (Number of amino acid substitutions/site) were computed using the Poisson correction method.

### Functional divergence

To evaluate the potential functional divergence and to predict the amino acid residues accounting for the functional differences among the three enzymes, Type I functional divergence was estimated using DIVERGE 2.0. Gaucher et al. [14] Sequences were classified into three different groups (IPCS, SPL and SPT) using the P-distance method (Additional file 1: Table S2). For each pairwise comparison, the coefficient of evolutionary functional divergence (θ) and standard error were determined. Utilizing the coefficient of evolutionary functional divergence (θ) the sequences were subjected to significant functional divergence and likelihood ratio test (LRT). Based on the site-specific posterior probabilities, sites experiencing functional divergence in the subgroups were identified [15]*,*[16].

### SDP (Specificity determining positions) analysis

SDP’s determine the differences in functional specificity within the protein family [17,18]. The input set of sequences was divided into 4 groups (IPCS, SMS, SPL and SPT) containing 34, 20, 40 and 78 sequences respectively. SDP’S were predicted by SDPfox and SDPpred and the Z scores were calculated for each alignment column.

### Selection pressure assessment of the protein families

The selected clades from functional divergence were submitted as nucleotide alignment in fasta format to the Selecton server for analysis of the non-synonymous (dN) versus synonymous substitution (dS) ratio [19]. To study the effect of selection pressure on the conservedness of the three proteins, the protein MSA was submitted to the CONSURF server, which classifies the residues based on their conservation in the MSA [20].

### Selection pressure assessment at the codon level

Selection pressure on the codon was estimated for the targeted organism *Leishmania*. Protozoan coding sequences were obtained from GenBank (release 137). In order to normalize codon usage within datasets of differing amino acid compositions, relative synonymous codon usage (RSCU) values were calculated. The reference set consisted of highly expressed genes pertaining to the lipid metabolism as reported by [21] Codon Adaptation Index (CAI) was calculated using CAI Calculator 2 [22]. Other species non-specific indices like ENc (Effective Number of Codon) and Fop (Frequency of optimized codons) were calculated using DAMBE software [23]) and CodonW respectively [24]. GC3s values were calculated using CodonO webserver [25]. Coding sequences of elongation factors were retrieved from GeneDB. A comparison of the CAI values of the elongation factors with the CAI values of IPCS, SPT and SPL was made to determine the codon usage bias in each of the three genes.

## Results and discussion

### Phylogenetic tree construction for the sphingolipid metabolism

**Figure 2:**
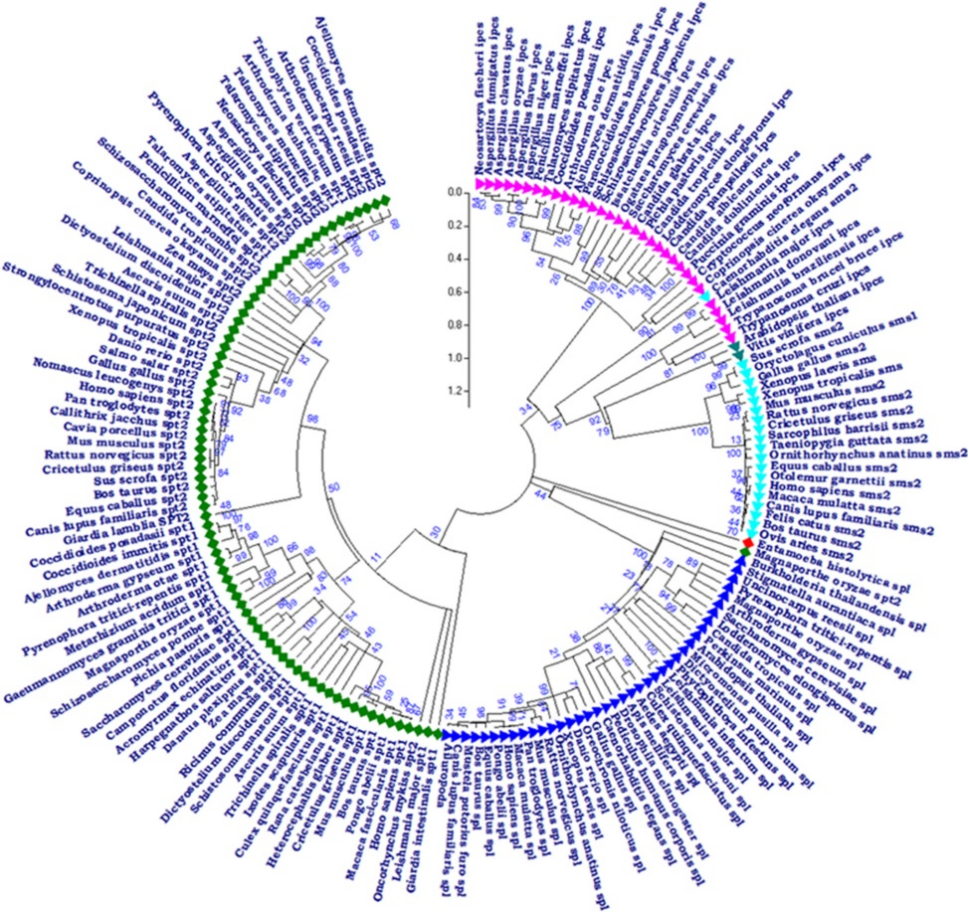
**The phylogenetic tree depicting the evolutionary relationship of the three enzymes (IPCS, SPL and SPT) belonging to the sphingolipid metabolism in comparison to SMS enzyme in the host.** The tree was constructed by the NJ method using MEGA5 with 10000 replicates. Bootstrap values are indicated on the branches, depicting the evolutionary relationship among the three protein families.

Phylogenetic analysis provides a basis for understanding the diversity within the conserved protein families. Phylogenetic tree for the sphingolipid metabolism included a total of 172 protein sequences belonging to the four protein families. The optimal tree was obtained and the sum of branch length was 46.11 (Figure 2). The rate of amino acid substitutions per site was 0.2. From the phylogenetic tree, it was observed that IPCS and SPL protein families were phylogenetically distant from SPT protein family. IPCS in *Leishmania* showed resemblance to the IPCS protein in plants as compared to other fungal groups.

### Functional divergence

**Figure 3:**
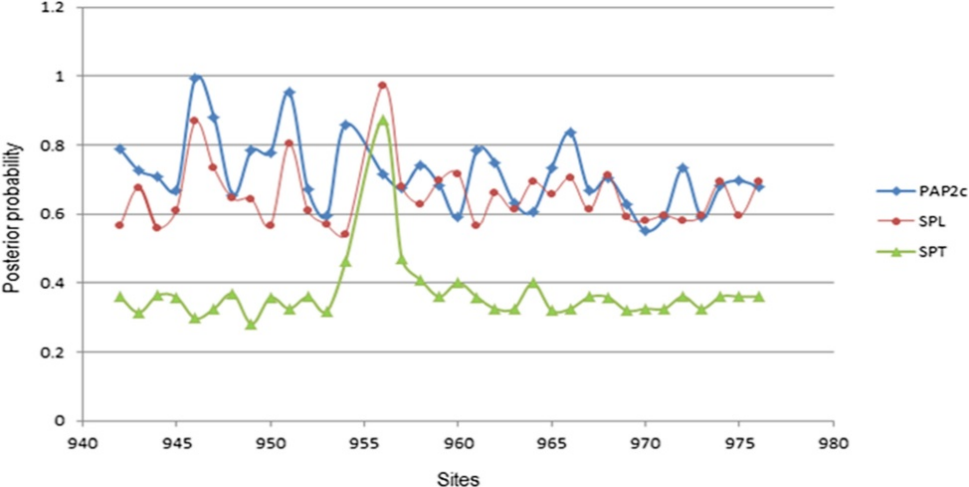
Sites with altered evolutionary rates identified by posterior probability along with functional divergence.

Phylogenetic analysis doesn’t provide much information for genes that have diverged by accumulating only few mutations. For such genes, analysis of the Type I functional divergence provides patterns of sequence variations across the gene family. Site specific functional divergence of a gene across a set of homologous gene sequences can be analyzed using Type-I functional divergence. Functional divergence correlates with the kind of selection pressure operating over the protein (positive, neutral or purifying), defining the extent of amino acid conservation. Evolutionary pressures operating over the parasitic proteins vary from one amino acid to another. Amino acids that are of functional importance, are evolutionary conserved, on the other hand amino acids experiencing high rates of sequence divergence alter the basic function of a protein. The coefficient of functional divergence for the PAP2c (0.69) and SPL (0.36) protein families was much higher than that of SPT (0.36) protein family (Figure 3). Site specific posterior probability analysis indicated that PAP2c and SPL protein families diverge from the SPT family.

### Specificity determining positions [SDP]

Gene duplication, deletion events along with the evolutionary selection pressures alter the basic biochemical properties of a protein like ligand binding, protein-protein interactions and the specificity towards the substrate. The amino acid positions which vary only in certain subgroups and alter the specificity of a protein are called the Specificity determining positions (SDP’s). Alignment positions, accounting for such functional specificity of the three enzymes in *L. major* were mapped and analyzed for their conservation. A total of 34 positions were identified as SDP’s in all the four groups (Additional file 1: Table S3).

### Selection pressure over the protein families

**Figure 4:**
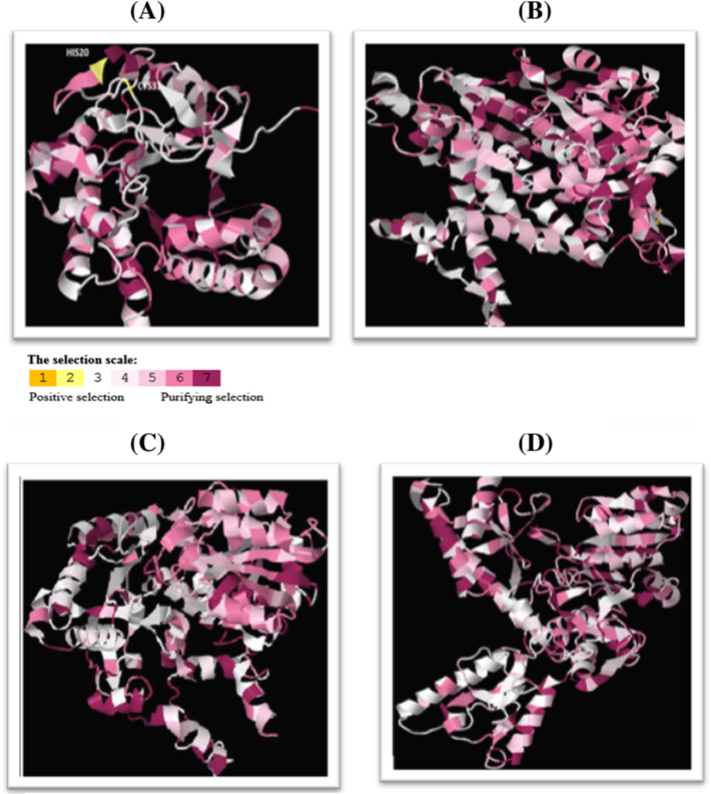
**Conservation profile of the protein families. A)** IPCS protein family **B)** SPL protein family **C)** SPT1 protein family **D)** SPT2 protein family. Residues marked in yellow indicate the presence of positive selection while those coloured in gray or pink indicate neutral and purifying selection respectively.

Selection pressure operating over protein families can be estimated using the ratio of non-synonymous substitutions and the synonymous substitutions. The extent of positive selection pressure, determines the relative rate of mutability of any amino acid (dN/dS ratio > 1). Purifying selection on the other hand, promotes conservation of the amino acids (DN/DS ratio < 1). Sphingolipid metabolism of parasites remains highly conserved among several parasites. As evident from the CONSURF analysis, most of the amino acids of all the three protein families fall under the effect of purifying selection and are functionally conserved across closely related species (Additional file 1: Figure S1A-D, Figure 4A-D). Amino acids in the IPCS protein family were less conserved as compared to SPL and SPT protein families. Two sites (HIS20 and CYS30) in the IPCS protein were under the influence of positive selection (Figure 4A). Relative mutability of such sites is much higher and hence inhibitor designing against the binding pockets containing these residues should be avoided in order to cut down the probability of developing drug resistance.

**Figure 5:**
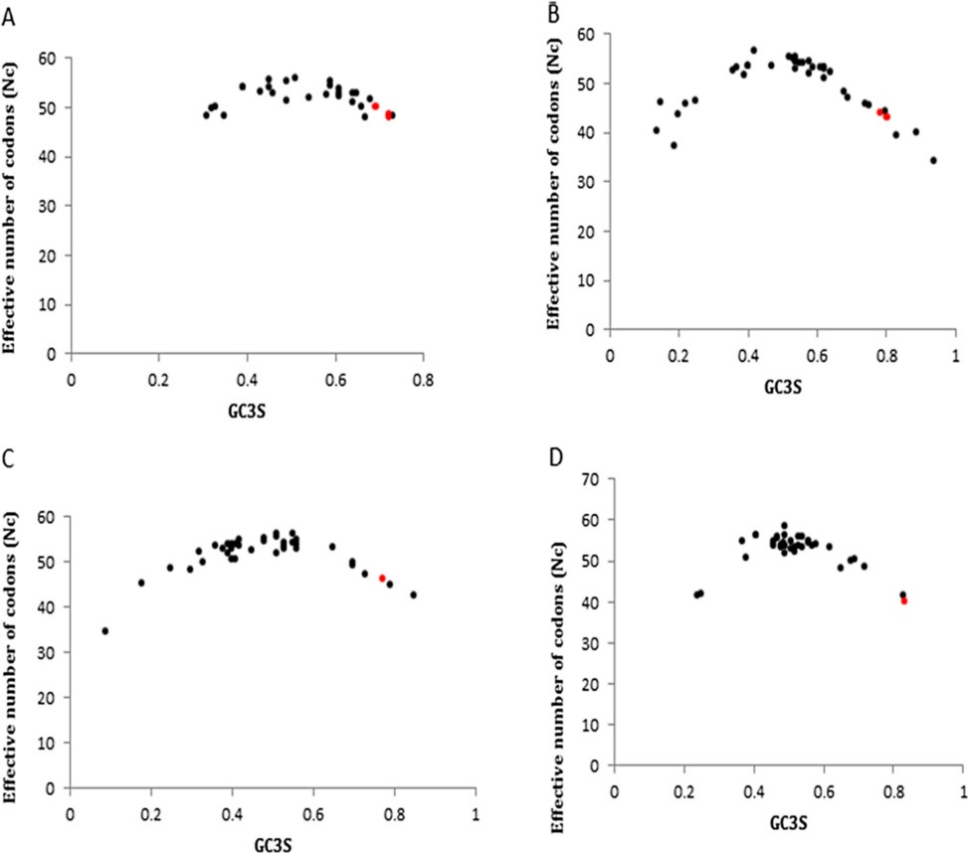
**ENc vs GC3s plot for A) IPCS protein family B) SPL protein family C) SPT1 protein family and D) SPT2 protein family.** Red dots represent the ENc and GC3s values of different Leishmania species. Lower ENc values together with the high GC3s value indicated the effect of selection pressure over the parasitic proteins.

Studies of synonymous codon usage also reveal information about the molecular evolution of individual genes. A gene can be characterized not only by its amino acid sequence but also by its codon usage shaped up by the balance between mutational bias and natural selection. Differences in the codon bias between species arise as a consequence of the selection pressure, which results in the non-uniform usage of synonymous codons within a gene. Bias can be either codon specific or gene specific. Optimal codons can also be defined by their nucleotide chemistry (GC content) and the codon usage bias. Genes display a non-random usage of synonymous codons and a measure of this non-randomness is RSCU (Relative synonymous codon usage). Codons with an RSCU value higher than 1 are used more frequently than expected, while codons with an RSCU value less than 1 indicate a lesser preference of the particular codon [26]. To understand the codon usage pattern for IPCS, SPL and SPT protein families, RSCU values were calculated and the codon adaptability index (CAI) was obtained [27]. CAI values of IPCS gene were slightly lesser than that of SPT or SPL. At least one of the codons for each of the three genes had a RSCU value greater than 1. We compared the ENc values of genes in *Leishmania* with that of other homologues, ENc values of *Leishmania* species were lesser than other organisms. This indicates the possibility of selection pressure, guiding the translational selection of optimal codons in *Leishmania*[28]. A plot of ENc vs GC3s indicated that the ENc values generally showed negative correlation with the GC content. All the genes tested in *Leishmania* had a higher predominance of G and C ending codons. Moreover GC content at the third synonymous position in *Leishmania* species was higher than that of other species. Codon usage pattern of all the genes therefore appears to be determined by the GC3s value (Figure 5). Higher GC content, lower Fop values along with lower ENc values suggest that there exists a moderate bias in the usage of synonymous codons in different *Leishmania* species. Codon usage bias was much more predominant in the SPT and SPL genes as compared to IPCS (Table 1).

**Table 1:** Codon usage bias indices for IPCS, SPL and SPT genes

**Enzyme name**	**Species**	**CAI**	**ENc**	**GC3S**	**FOP**	**Highest RSCU**
IPCS	*L.major*	0.604	50.34	0.72	0.362	UUG (Leu)
	*L.donovani*	0.623	48.61	0.70	0.385	CGC (Arg)
	*L.braziliensis*	0.573	48.21	0.69	0.394	CGC (Arg)
SPL	*L.major*	0.681	44.22	0.78	0.39	CUG (Leu)
	*L.infactum*	0.685	43.26	0.80	0.37	CGC (Arg)
SPT-1	*L.major*	0.694	46.45	0.77	0.323	CUG (Leu)
SPT-2	*L.major*	0.720	40.29	0.83	0.373	GUU (Val)

RSCU (Relative synonymous codon usage was calculated to estimate the number of cotton under codon usage bias. CUB indices calculate are a) CAI – Codon adaptability index b) ENc – Effective number of codons c) GC3S – GC content at third synonymous position d) FOP – Frequency of optimized codons. A comparison of these indices between *Leishmania* and other homologous species is made. Range of indices between homologous species is given in the brackets. IPCS, SPT and SPL genes of Leishmania experience higher codon usage bias as compared to their homologues.

Elongation factors show higher expression and stronger codon usage bias. A comparison of the CAI values of the various elongation factors of Leishmania with the genes coding for our target proteins was done. The CAI value of SPT gene was comparable with that of CAI values of elongation factors. SPT gene therefore showed stronger codon usage bias than SPL or IPCS (Table 2). In due consideration of the role of selection pressure deciding the evolvability of target proteins, it can be concluded that the three target proteins were moderately conserved and given their importance in the sphingolipid metabolism, these proteins could serve as good drug targets.

**Table 2:** CAI values of IPCS, SPL and SPT genes in comparison with CAI values of the highly expressed elongation factors of Leishmania

**Enzyme**	**Species**	**CAI**	**Elongation factor**	**CAI**
IPCS	*L.major*	0.604	eEF1B beta 1 (*LmjF.34.0820*)	0.866
	*L.donovani*	0.623		
	*L.braziliensis*	0.573		
SPL	*L.major*	0.681	EF2-1 (*LmjF.36.0180)*	0.821
	*L.infactum*	0.685		
SPT-1	*L.major*	0.694	EF1G (*LmjF.09.0970)*	0.847
SPT-2	*L.major*	0.720	EF-Tu (*LmjF.18.0740)*	0.768
			EF1A (*LmjF.17.0081)*	0.880

SPT gene showed a higher CAI value as compared to IPCS and SPL genes.

## Conclusion

The key enzymes (IPCS, SPL and SPT) of the sphingolipid metabolism of *L.major* were studied in relation to other members of the same protein family. The present study supports the classification of sphingolipid metabolism with a high bootstrap value on the internal branches. To derive sufficient information about the kind of evolutionary pressure the three enzymes are being subjected to, functional divergence and conservation at the level of both amino acids and codons was studied. Functional divergence analysis indicated that PAP2c family diverged from SPL and SPT. Amino acids accounting for functional specificity (SDP’s) of the three enzymes in *L.major* were mapped. Along with the functional divergence, the selection pressures over the protein families were assessed. The effect of selection pressure was more predominant at the codon level as indicated by the CUB indices. IPCS gene showed a lower codon usage bias as compared to SPL and SPT genes. At the protein level, the effect of purifying selection was largely predominant and this accounted for the functional conservation of the three drug targets.

## Abbreviations

ER: Endoplasmic reticulum; IPCS: Inositol phosphoryl ceramide synthase; SPL: Sphingosine 1-P-lyase; SPT1: Serine palmitoyl transferase 1; SPT2: Serine palmitoyl transferase 2; SMS: Sphingomyelin synthase; NJ: Neighbour-joining; ME: Minimum evolution; SDP: Specificity determining positions; RSCU: Relative synonymous codon usage; ENc: Effective number of codon; Fop: Frequency of optimized codons; CAI: Codon adaptation index; CDS: Coding sequence; MSA: Multiple sequence alignment; CUB: Codon usage bias.

## Competing interests

The authors declare that they have no competing interests*.*

## Authors’ contribution

Conceived and designed the experiments: SHS, SOS, VM. SOS and VM participated in the acquisition of sequences. SOS, VM participated in the phylogenetic tree construction and functional divergence analysis. VM SOS contributed in the selection pressure assessment of the protein families. SHS VM SOS contributed to the data analysis. SHS, VM, SOS wrote the manuscript. All the authors read and approve the final manuscript.

## Supplementary Material

Additional file 1 Supplementary file. Click here for file
