# Supplementary material for: Molecular evolution of the enzymes involved in the sphingolipid metabolism of Leishmania: selection pressure in relation to functional divergence and conservation
Source: BMC Evol Biol. 2014 Jun 21;14:142. doi: 10.1186/1471-2148-14-142 (PMC4092354; doi:10.1186/1471-2148-14-142)
Supplement: Additional file 1 — Supplementary file. [file 1471-2148-14-142-S1.docx]

**SUPPLEMENTARY FILE**

**Supplementary file**

**Accession numbers**

**SPL PROTEIN FAMILY**

| **Sr. No.** | **Organism Name** | **Accession No.** | **Length of the sequence** |
| --- | --- | --- | --- |
|  | *Pan troglodytes_spl* | XP_521504.2 | 568aa |
|  | *Nomascus leucogenys_spl* | XP_003271253.1 | 566aa |
|  | *Pongo abelii_spl* | ref\|NP_001126976.1 | 568aa |
|  | *Macaca mulatta_spl* | ref\|XP_001106861.2 | 565aa |
|  | *Equus caballus_spl* | ref\|XP_001502842.1 | 568aa |
|  | *Ailuropoda melanoleuca_spl* | ref\|XP_002913763.1 | 568aa |
|  | *Canis lupus familiaris_spl* | ref\|XP_546150.2 | 568aa |
|  | *Mustela putorius furo_spl* | gb\|AES06466.1 | 568aa |
|  | *Bos taurus_spl* | ref\|NP_001091522.1 | 568aa |
|  | *Rattus norvegicus_spl* | ref\|NP_775139.1 | 568aa |
|  | *Mus musculus_spl* | ref\|NP_033189.2 | 568aa |
|  | *Gallus gallus_spl* | ref\|NP_001007947.1 | 561aa |
|  | *Danio rerio_spl* | ref\|NP_001082938.1 | 572aa |
|  | *Xenopus laevis_spl* | ref\|NP_001091225.1 | 453aa |
|  | *Aedes aegypti_spl* | ref\|XP_001656538.1 | 538aa |
|  | *Culex quinquefasciatus_spl* | ref\|XP_001868402.1 | 539aa |
|  | *Schistosoma mansoni_spl* | ref\|XP_002577380.1 | 1239aa |
|  | *Caenorhabditis elegans_spl* | ref\|NP_499913.1 | 552aa |
|  | *Homo sapiens_spl* | NP_003892.2 | 567aa |
|  | *Drosophila melanogaster_spl* | NP_652032.1 | 545aa |
|  | *Dictyostelium discoideum_spl* | XP_639378.1 |  |
|  | *Saccharomyces cerevisiae_spl (strain ATCC 204508 / S288c)* | NP_010580.1 | 589aa |
|  | *Arabidopsis thaliana_spl* | NP_174119.1 | 544aa |
|  | *Burkholderia thailandensis_spl* | YP_438510.1 | 473aa |
|  | *Uncinocarpus reesii_spl* | XP_002584454.1 | 571aa |
|  | *Magnaporthe oryzae_spl* | XP_003715360.1 |  |
|  | *Lodderomyces elongisporus_spl* | XP_001526246.1 | 584aa |
|  | *Leishmania infantum JPCM5* | XP_001467082.1 | 537aa |
|  | *Leishmania major strain Friedlin* | XP_001684840.1 | 537aa |
|  | *Oreochromis niloticus* | XP_003441274.1 | 565aa |
|  | *Ornithorhynchus anatinus* | XP_001508856.2 | 611aa |
|  | *Apis mellifera* | XP_623988.1 | 549aa |
|  | *Pediculus humanus corporis* | XP_002429236.1 | 554aa |
|  | *Stigmatella aurantiaca* | EC:4.1.2.27 | 440aa |
|  | *Candida tropicalis MYA-3404* | XP_002548177.1 | 596aa |
|  | *Dictyostelium purpureum* | XP_003284082.1 | 531aa |
|  | *Micromonas pusilla CCMP1545* | XP_003060737.1 | 561aa |
|  | *Entamoeba histolytica HM-1:IMSS* | XP_001913761.1 | 110aa |
|  | *Phytophthora infestans T30-4* | XP_002908984.1 | 607aa |
|  | *Perkinsus marinus ATCC 50983* | XP_002783187.1 | 535aa |

**(1A)**

**SPT1 PROTEIN FAMILY**

| **Sr. No.** | **Organism Name** | **Accession No.** |
| --- | --- | --- |
| 1 | *Saccharomyces cerevisiae_spt1 (strain ATCC 204508 / S288c) (Baker's yeast)* | NP_014025.1 |
| 2 | *Homo sapiens_spt1 (Human)* | NP_006406.1 |
| 3 | *Mus musculus_spt1 (mouse)* | NP_033295.2 |
| 4 | *Schizosaccharomyces pombe_spt1 (strain 972 / ATCC 24843) (Fission yeast)* | NP_595848.1 |
| 5 | *Pichia pastoris_spt1 (strain GS115 / ATCC 20864) (Yeast)* | XP_002490853.1 |
| 6 | *Coccidioides immitis_spt1 (strain RS) (Valley fever fungus)* | XP_001247983.1 |
| 7 | *Trichinella spiralis_spt1 (Trichina worm)* | XP_003374112.1 |
| 8 | *Cricetulus griseus_spt1 (Chinese hamster) (Cricetulus barabensis griseus)* | NP_001233688.1 |
| 9 | *Dictyostelium discoideum_spt1 (Slime mold)* | XP_647518.1 |
| 10 | *Neosartorya fumigata_spt1 (strain CEA10 / CBS 144.89 / FGSC A1163) (Aspergillus fumigatus)* | EDP47868.1 |
| 11 | *Arthroderma otae_spt1 (strain ATCC MYA-4605 / CBS 113480) (Microsporum canis)* | XP_002847737.1 |
| 12 | *Arthroderma benhamiae_spt1 (strain ATCC MYA-4681 / CBS 112371) (Trichophyton mentagrophytes)* | XP_003017462.1 |
| 13 | *Trichophyton verrucosum_spt1 (strain HKI 0517)* | XP_003022680.1 |
| 14 | *Arthroderma gypseum_spt1 (strain ATCC MYA-4604 / CBS 118893) (Microsporum gypseum)* | XP_003176313.1 |
| 15 | *Acromyrmex echinatior_spt1 (Panamanian leafcutter ant) (Acromyrmex octospinosus echinatior)* | EGI57899.1 |
| 16 | *Danaus plexippus_spt1 (Monarch butterfly)* | EHJ75593.1 |
| 17 | *Pan troglodytes_spt1 (Chimpanzee)* | BAK63921.1 |
| 18 | *Magnaporthe oryzae_spt1 (strain 70-15 / ATCC MYA-4617 / FGSC 8958) (Rice blast fungus) (Pyricularia oryzae)* | XP_003718108.1 |
| 19 | *Culex quinquefasciatus_spt1 (Southern house mosquito) (Culex pungens)* | XP_001847608.1 |
| 20 | *Heterocephalus glaber_spt1 (Naked mole rat)* | EHB00583.1 |
| 21 | *Bos taurus_spt1 (Bovine)* | NP_001029921.1 |
| 22 | *Pongo abelii_spt1 (Sumatran orangutan) (Pongo pygmaeus abelii)* | Q5R9T5.1 |
| 23 | *Macaca fascicularis_spt1 (Crab-eating macaque) (Cynomolgus monkey)* | Q60HD1.1 |
| 24 | *Metarhizium acridum_spt1 (strain CQMa 102)* | EFY92418.1 |
| 25 | *Metarhizium robertsii_spt1 (strain ARSEF 23 / ATCC MYA-3075) (Metarhizium anisopliae)* | EFY99528.1 |
| 26 | *Harpegnathos saltator_spt1 (Jerdon's jumping ant)* | EFN80030.1 |
| 27 | *Giardia intestinalis_spt1 (strain ATCC 50581 / GS clone H7) (Giardia lamblia)* | EET01901.1 |
| 28 | *Camponotus floridanus_spt1 (Florida carpenter ant)* | EFN68827.1 |
| 29 | *Schistosoma mansoni_spt1 (Blood fluke)* | XP_002574251.1 |
| 30 | *Ricinus communis_spt1 (Castor bean)* | XP_002518026.1 |
| 31 | *Penicillium marneffei_spt1 (strain ATCC 18224 / CBS 334.59 / QM 7333)* | XP_002153066.1 |
| 32 | *Ajellomyces dermatitidis_spt1 (strain SLH14081) (Blastomyces dermatitidis)* | XP_002629107.1 |
| 33 | *Ascaris suum_spt1 (Pig roundworm) (Ascaris lumbricoides)* | ADY43486.1 |
| 34 | *Zea mays_spt1 (Maize)* | NP_001147940.1 |
| 35 | *Pyrenophora tritici-repentis_spt1 (strain Pt-1C-BFP) (Wheat tan spot fungus) (Drechslera tritici-repentis)* | XP_001936644.1 |
| 36 | *Wuchereria bancrofti_spt1* | EJW77119.1 |
| 37 | *Leishmania major_spt1* | XP_001686478.1 |
| 38 | *Ixodes scapularis_spt1 (Black-legged tick) (Deer tick)* | XP_002433636.1 |
| 39 | *Pichia angusta_spt1 (strain ATCC 26012 / NRRL Y-7560 / DL-1) (Yeast) (Hansenula polymorpha)* |  |
| 40 | *Lithobates catesbeiana_spt1 (American bullfrog) (Rana catesbeiana)* | ACO51554.1 |
| 41 | *Coccidioides posadasii_spt1 (strain RMSCC 757 / Silveira) (Valley fever fungus)* | EFW13413.1 |
| 42 | *Gaeumannomyces graminis var. tritici_spt1 (strain R3-111a-1) (Wheat and barley take-all root rot fungus)* | EJT75801.1 |
| 43 | *Talaromyces stipitatus_spt1 (strain ATCC 10500 / CBS 375.48 / QM 6759 / NRRL 1006) (Penicillium stipitatum)* | XP_002487531.1 |

**(1B)**

**SPT2 PROTEIN FAMILY**

| **Sr. No.** | **Organism Name** | **Accession No.** |
| --- | --- | --- |
| 1 | *Homo sapiens_spt2* | NP_004854.1 |
| 2 | *Pan troglodytes_spt2* | XP_510095.3 |
| 3 | *Nomascus leucogenys_spt2* | XP_003260859.1 |
| 4 | *Cavia porcellus_spt2* | XP_003472501.1 |
| 5 | *Mus musculus_spt2* | NP_035609.1 |
| 6 | *Rattus norvegicus_spt2* | NP_001032174.1 |
| 7 | *Callithrix jacchus_spt2* | XP_002754273 |
| 8 | *Cricetulus griseus_spt2* | NP_001233609.1 |
| 9 | *Sus scrofa_spt2* | XP_001926625.4 |
| 10 | *Bos taurus_spt2* | NP_001092551.1 |
| 11 | *Equus caballus_spt2* | XP_001492046.1 |
| 12 | *Gallus gallus_spt2* | NP_001006483.1 |
| 13 | *Salmo salar_spt2* | gb\|ACI68064.1 |
| 14 | *Oncorhynchus mykiss_spt2* | gb\|ACO08449.1 |
| 15 | *Xenopus silurana_spt2* | XP_002938915.1 |
| 16 | *Strongylocentrotus purpuratus_spt2* | XP_786894.3 |
| 17 | *Trichiniella spiralis_spt2* | XP_003375594.1 |
| 18 | *Ascaris suum_spt2* | ADY41739.1 |
| 19 | *Zea Mays_spt2* | NP_001148953.1 |
| 20 | *Danio rerio_spt2* | NP_001018455.1 |
| 21 | *Talaromyces stipitatus_spt2* | XP_002484864.1 |
| 22 | *Aspergillus niger_spt2* | XP_001398841.2 |
| 23 | *Neosartorya fumigata_spt2 (Aspergillus fumigatus)* | XP_001264572.1 |
| 24 | *Arthroderma otae_spt2* | XP_002845325.1 |
| 25 | *Arthroderma gypseum_spt2* | XP_003171686.1 |
| 26 | *Ajellomyces dermatitidis_spt2* | XP_002620119.1 |
| 27 | *Coccidioides posadasii_spt2* | XP_003067044.1 |
| 28 | *Uncinocarpus reesii_spt2* | XP_002543814 |
| 29 | *Pyrenophora tritici repentis_spt2* | XP_001936333.1 |
| 30 | *Magnaporthe oryzae_spt2* | XP_003713199.1 |
| 31 | *Aspergillus oryzae_spt2* | XP_001818901.1 |
| 32 | *Leishmania major_spt2* | AAO92019.1 |
| 33 | *Schizosaccharomyces pombe_spt2* | XP_001713103.1 |
| 34 | *Candida tropicalis_spt2* | XP_002549375.1 |
| 35 | *Coprinopsis cineria okayama_spt2* | XP_001837771.2 |
| 36 | *Dictyostelium discoideum_spt2* | XP_635115.1 |
| 37 | *Aspergillus flavus_spt2* | XP_002380337.1 |
| 38 | *Giardia lamblia_spt2* | XP_001704960.1 |
| 39 | *Penicillium marneffe_spt2i* | XP_002149297.1 |
| 40 | *Schistosoma japonicum_spt2* | CAX72889 |

**(1C)**

**IPCS PROTEIN FAMILY**

| **Sr. No.** | **Organism Name** | **Accession No.** | **Length of the sequence** |
| --- | --- | --- | --- |
| 1 | *Pichia pastoris_ipcs* | XP_002492322.1 | 378aa |
| 2 | *Saccharomyces cerevisiae_ipcs* | [NP_012922.1](http://www.ncbi.nlm.nih.gov/protein/6322849?report=genbank&log$=prottop&blast_rank=1&RID=E2AFM8HF016) | 401aa |
| 3 | *Candida albicans_ipcs* | [XP_715708.1](http://www.ncbi.nlm.nih.gov/protein/68480652?report=genbank&log$=prottop&blast_rank=1&RID=E2AZ6NDH01R) | 471aa |
| 4 | *Trypanosoma_brucei_bruce_ipcs* | [XP_827247.1](http://www.ncbi.nlm.nih.gov/protein/71745234?report=genbank&log$=prottop&blast_rank=1&RID=E2B2KASD01R) | 355aa |
| 5 | *Leishmania_major_ipcs* | [XP_003722892.1](http://www.ncbi.nlm.nih.gov/protein/389595339?report=genbank&log$=prottop&blast_rank=1&RID=E2B35ST0016) | 338aa |
| 6 | *Schizosaccharomyces_pombe_ipcs* | NP_592999.1 | 422aa |
| 7 | *Trypanosoma_cruzi_ipcs* | [XP_821506.1](http://www.ncbi.nlm.nih.gov/protein/71659570?report=genbank&log$=prottop&blast_rank=1&RID=E2B65JSU016) | 335aa |
| 8 | *Candida_glabrata_ipcs* | [XP_448347.1](http://www.ncbi.nlm.nih.gov/protein/50291829?report=genbank&log$=prottop&blast_rank=1&RID=E2B6R8WW01R) | 416aa |
| 9 | *Cryptococcus_neoformans_ipcs* | [AAD28749.1](http://www.ncbi.nlm.nih.gov/protein/4741889?report=genbank&log$=prottop&blast_rank=1&RID=E2BCXCSA016) | 465aa |
| 10 | *Candida_tropicalis_ipcs* | [AAD28748.2](http://www.ncbi.nlm.nih.gov/protein/7144496?report=genbank&log$=prottop&blast_rank=1&RID=E2BDYKDT016) | 458aa |
| 11 | *Candida_dubliniensis_ipcs* | [XP_002420413.1](http://www.ncbi.nlm.nih.gov/protein/241955385?report=genbank&log$=prottop&blast_rank=1&RID=E2BJD372016) | 476aa |
| 12 | *Candida_parapsilosis_ipcs* | [AAD28747.1](http://www.ncbi.nlm.nih.gov/protein/4741885?report=genbank&log$=prottop&blast_rank=1&RID=E2BKFG1A016) | 505aa |
| 13 | *Arabidopsis_thaliana_ipcs* | [NP_190970.1](http://www.ncbi.nlm.nih.gov/protein/15232394?report=genbank&log$=prottop&blast_rank=1&RID=E2BT6W4701R) | 305aa |
| 14 | *Vitis_vinifera_ipcs* | [XP_002276211.1](http://www.ncbi.nlm.nih.gov/protein/225440552?report=genbank&log$=prottop&blast_rank=1&RID=E2BMDRD6016) | 315aa |
| 15 | *Ogataea_parapolymorpha_ipcs* | [EFW97988.1](http://www.ncbi.nlm.nih.gov/protein/320583775?report=genbank&log$=prottop&blast_rank=1&RID=E2C1GEH2016) | 416aa |
| 16 | *Leishmania_donovani_ipcs* | [XP_003865080.1](http://www.ncbi.nlm.nih.gov/protein/398023837?report=genbank&log$=prottop&blast_rank=1&RID=E2C1UNEE016) | 385aa |
| 17 | *Leishmania_braziliensis_ipcs* | [XP_001568591.1](http://www.ncbi.nlm.nih.gov/protein/154345299?report=genbank&log$=prottop&blast_rank=1&RID=E2CB31AV01R) | 385aa |
| 18 | *Aspergillus_oryzae_ipcs* | [XP_001819689.1](http://www.ncbi.nlm.nih.gov/protein/169770439?report=genbank&log$=prottop&blast_rank=1&RID=E2C6RW0C01R) | 439aa |
| 19 | *Coccidioides_posadasii_ipcs* | [XP_003068380.1](http://www.ncbi.nlm.nih.gov/protein/303316756?report=genbank&log$=prottop&blast_rank=1&RID=E2C74WH901R) | 438aa |
| 20 | *Arthroderma_otae_ipcs* | [XP_002847944.1](http://www.ncbi.nlm.nih.gov/protein/296815214?report=genbank&log$=prottop&blast_rank=1&RID=E2CDA8YV016) | 402aa |
| 21 | *Ajellomyces_dermatitidis_ipcs* | [XP_002627820.1](http://www.ncbi.nlm.nih.gov/protein/261206166?report=genbank&log$=prottop&blast_rank=1&RID=E2CGRJK201R) | 438aa |
| 22 | *Penicillium_marneffei_ipcs* | [XP_002144251.1](http://www.ncbi.nlm.nih.gov/protein/212528188?report=genbank&log$=prottop&blast_rank=1&RID=E2CH1VR201R) | 445aa |
| 23 | *Neosartorya_fischeri_ipcs* | [XP_001263463.1](http://www.ncbi.nlm.nih.gov/protein/119491937?report=genbank&log$=prottop&blast_rank=1&RID=E2CHFMZW016) | 436aa |
| 24 | *Talaromyces_stipitatus_ipcs* | [XP_002341288.1](http://www.ncbi.nlm.nih.gov/protein/242767024?report=genbank&log$=prottop&blast_rank=1&RID=E2CHTM1T016) | 437aa |
| 25 | *Coprinopsis_cinerea_okayama_ipcs* | [XP_001838243.1](http://www.ncbi.nlm.nih.gov/protein/169863242?report=genbank&log$=prottop&blast_rank=1&RID=E2CVV7KJ01R) | 419aa |
| 26 | *Paracoccidioides_brasiliensis_ipcs* | [EEH17329.1](http://www.ncbi.nlm.nih.gov/protein/225679045?report=genbank&log$=prottop&blast_rank=1&RID=E2CWSTBH016) | 438aa |
| 27 | *Aspergillus_niger_ipcs* | [XP_001397677.1](http://www.ncbi.nlm.nih.gov/protein/145252328?report=genbank&log$=prottop&blast_rank=1&RID=E2D28J0501R) | 439aa |
| 28 | *Aspergillus_fumigatus_ipcs* | [XP_754623.1](http://www.ncbi.nlm.nih.gov/protein/70999812?report=genbank&log$=prottop&blast_rank=1&RID=E2D2H8JB01R) | 436aa |
| 29 | *Aspergillus_clavatus_ipcs* | [XP_001270996.1](http://www.ncbi.nlm.nih.gov/protein/121705466?report=genbank&log$=prottop&blast_rank=1&RID=E2DDG2ME01R) | 436aa |
| 30 | *Schizosaccharomyces_japonicus_ipcs* | [XP_002171784.1](http://www.ncbi.nlm.nih.gov/protein/213402023?report=genbank&log$=prottop&blast_rank=1&RID=E2DDUXST01R) | 422aa |
| 31 | *Aspergillus_flavus_ipcs* | [XP_001819689.1](http://www.ncbi.nlm.nih.gov/protein/169770439?report=genbank&log$=prottop&blast_rank=1&RID=E2DE38RJ01R) | 439aa |
| 32 | *Lodderomyces_elongisporus_ipcs* | [XP_001524762.1](http://www.ncbi.nlm.nih.gov/protein/149237771?report=genbank&log$=prottop&blast_rank=1&RID=E2DEEZ1R01R) | 354aa |
| 33 | *Puccinia_graminis_ipcs* | [XP_003333402.2](http://www.ncbi.nlm.nih.gov/protein/403174429?report=genbank&log$=prottop&blast_rank=1&RID=E2DHCJGZ016) | 443aa |

|  |  |  |
| --- | --- | --- |

**(1D)**

**SMS PROTEIN FAMILY**

| **S. No** | **Organism Name** | **Accession No.** | **Length of the sequence** |
| --- | --- | --- | --- |
| 1 | *Mus musculus_sms* | [NP_083219.2](http://www.ncbi.nlm.nih.gov/protein/27229211?report=genbank&log$=prottop&blast_rank=1&RID=E2DTBC5M01R) | 365aa |
| 2 | *Homo sapiens_sms* | NP_689834.1 | 365aa |
| 3 | *Rattus norvegicus_sms* | NP_001014065.1 | 365aa |
| 4 | *Sus scrofa_sms* | NP_001090907.1 | 413aa |
| 5 | *Gallus gallus_sms* | NP_001090907.1 | 413aa |
| 6 | *Cricetulus griseus_sms* | XP_003509690.1 | 365aa |
| 7 | *Canis lupus familiaris_sms* | XP_535684.2 | 365aa |
| 8 | *Sarcophilus harrisii_sms* | XP_003772964.1 | 365aa |
| 9 | *Bos taurus_sms* | NP_001192806.1 | 365aa |
| 10 | *Equus caballus_sms* | XP_001503622.1 | 365aa |
| 11 | *Ovis aries_sms* | XP_004009687.1 | 365aa |
| 12 | *Felis catus_sms* | XP_003985150.1 | 365aa |
| 13 | *Macaca mulatta_sms* | XP_001084598 | 365aa |
| 14 | *Otolemur garnettii_sms* | XP_003796392.1 | 365aa |
| 15 | *Taeniopygia guttata_sms* | XP_002196898.1 | 365aa |
| 16 | *Ornithorhynchus anatinus_sms* | XP_001511130.1 | 365aa |
| 17 | *Oryctolagus cuniculus_sms* | XP_002718537.1 | 419aa |
| 18 | *Caenorhabditis elegans_sms* | NP_508182.1 | 315aa |
| 19 | *Xenopus laevis_sms* | NP_001084610.1 | 412aa |
| 20 | *Xenopus (Silurana) tropicalis_sms* | NP_001008197.1 | 412aa |

**(1E)**

**Table S1 A-E: Accession numbers for SPL, SPT1, SPT 2, IPCS and SMS enzymes included in the study**

| **S.No** | **Organism name** | **Accession No.** |
| --- | --- | --- |
| 1 | *Pan troglodytes_spl* | XP_521504.2 |
| 2 | *Pongo abelii_spl* | ref\|NP_001126976.1 |
| 3 | *Homo sapiens_spl* | NP_003892.2 |
| 4 | *Drosophila melanogaster_spl* | NP_652032.1 |
| 5 | *Canis lupus familiaris_spl* | ref\|XP_546150.2 |
| 6 | *Macaca mulatta_spl* | ref\|XP_001106861.2 |
| 7 | *Bos taurus_spl* | ref\|NP_001091522.1 |
| 8 | *Xenopus laevis_spl* | ref\|NP_001091225.1 |
| 9 | *Aedes aegypti_spl* | ref\|XP_001656538.1 |
| 10 | *Culex quinquefasciatus_spl* | ref\|XP_001868402.1 |
| 11 | *Schistosoma mansoni_spl* | ref\|XP_002577380.1 |
| 12 | *Burkholderia thailandensis_spl* | YP_438510.1 |
| 13 | *Saccharomyces cerevisiae_spl (strain ATCC 204508 / S288c)* | NP_010580.1 |
| 14 | *Apis mellifera spl* | XP_623988.1 |
| 15 | *Danio rerio_spl* | ref\|NP_001082938.1 |
| 16 | *Arabidopsis thaliana_spl* | NP_174119.1 |
| 17 | *L.infantum spl* | XP_001467082.1 |
| 18 | *L.major spl* | XP_001684840.1 |
| 19 | *Saccharomyces cerevisiae_spl (strain ATCC 204508 / S288c)* | NP_010580.1 |
| 20 | *Mus musculus_spt1 (mouse)* | NP_033295.2 |
| 21 | *Ixodes scapularis_spt1 (Black-legged tick) (Deer tick)* | XP_002433636.1 |
| 22 | *Homo sapiens_spt1 (Human)* | NP_006406.1 |
| 23 | *Schistosoma mansoni_spt1 (Blood fluke)* | XP_002574251.1 |
| 24 | *Culex quinquefasciatus_spt1 (Southern house mosquito) (Culex pungens)* | XP_001847608.1 |
| 25 | *Cricetulus griseus_spt1 (Chinese hamster) (Cricetulus barabensis griseus)* | NP_001233688.1 |
| 26 | *Trypanosoma_brucei_bruce_ipcs* | XP_827247.1 |
| 27 | *Leishmania_major_ipcs* | XP_003722892.1 |
| 28 | *Trypanosoma_cruzi_ipcs* | XP_821506.1 |
| 29 | *Leishmania_donovani_ipcs* | XP_003865080.1 |
| 30 | *Leishmania_braziliensis_ipcs* | XP_001568591.1 |
| 31 | *Mus musculus_sms* | NP_083219.2 |
| 32 | *Homo sapiens_sms* | NP_689834.1 |
| 33 | *Gallus gallus_sms* | NP_001090907.1 |
| 34 | *Equus caballus_sms* | XP_001503622.1 |
| 35 | *Ovis aries_sms* | XP_004009687.1 |
| 36 | *Xenopus (Silurana) tropicalis_sms* | NP_001008197.1 |

**Table S2: Accession numbers for organisms included for DIVERGE analysis**

| **Alignment position** | **SPL** | **IPCS** | **SPT** | **Z-score** |
| --- | --- | --- | --- | --- |
| 966 | RKS | YIF | TAC | 16.68 |
| 900 | G | VEI | STA | 14.9 |
| 1005 | INF | EKR | SAG | 14.34 |
| 925 | ENS | LIM | GAR | 13.89 |
| 903 | S | ATM | YFH | 13.36 |
| 1021 | V | PAS | KEN | 13.31 |
| 934 | APT | GRE | FED | 12.4 |
| 1083 | K | SIA | NPR | 11.97 |
| 1129 | GAN | MLI | DEH | 11.9 |
| 897 | TS | WST | DEH | 11.83 |
| 1113 | QPS | LFV | GDV | 11.74 |
| 1003 | D | WIL | HNC | 11.72 |
| 1069 | FW | EKA | IVL | 11.52 |
| 971 | NDR | GAY | IFL | 11.52 |
| 685 | GST | KTA | LTP | 11.38 |
| 905 | LMI | EGF | GNS | 11.16 |
| 909 | KLR | YLV | NSD | 11.07 |
| 1013 | VLK | ATL | STA | 11.05 |
| 902 | E | PHQ | NDS | 10.97 |
| 945 | MIA | ASP | ERD | 10.96 |
| 858 | DEG | ILF | CNA | 10.79 |
| 1095 | LMA | HLS | KRA | 10.74 |
| 943 | FLC | VIY | EDR | 10.43 |
| 975 | LIV | QDN | VSC | 10.43 |
| 861 | PGQ | WNL | RYV | 10.10 |
| 1107 | FTY | SCF | GRD | 10.09 |
| 924 | PSY | VFR | YSF | 10.03 |
| 917 | EAD | LKR | AVS | 9.87 |
| 1072 | KPD | GCH | EKP | 9.7 |
| 1163 | LIM | PGE | SHT | 9.63 |
| 1020 | H | SNG | FYH | 9.55 |
| 1007 | EAG | LVI | RVQ | 9.04 |
| 1014 | KRE | NDH | GRK | 8.94 |
| 849 | FAY | SRC | RVI | 8.83 |

**Table S3: Specificity determining positions in the alignment**

**
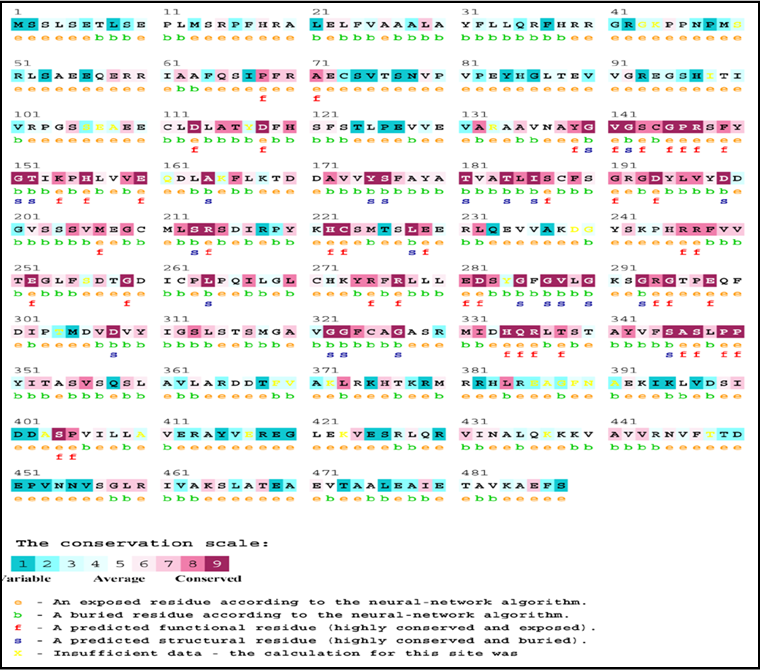

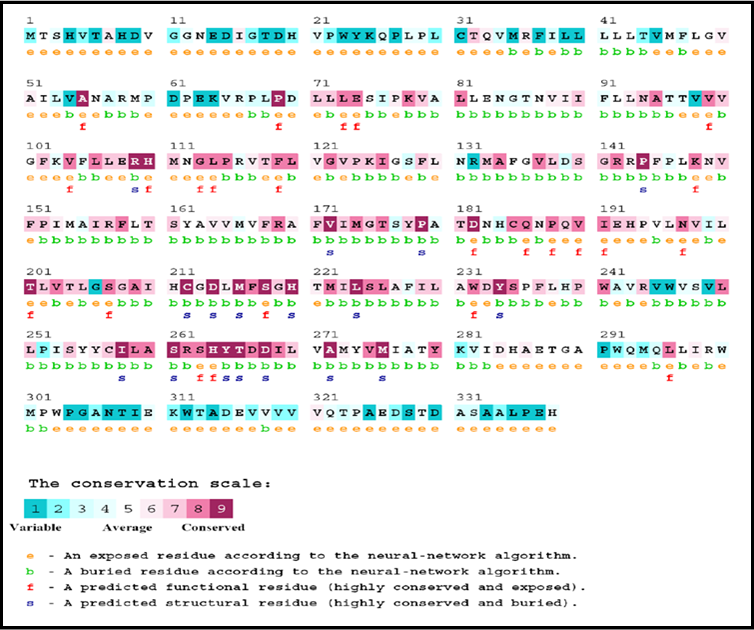
**

**(A)**

**
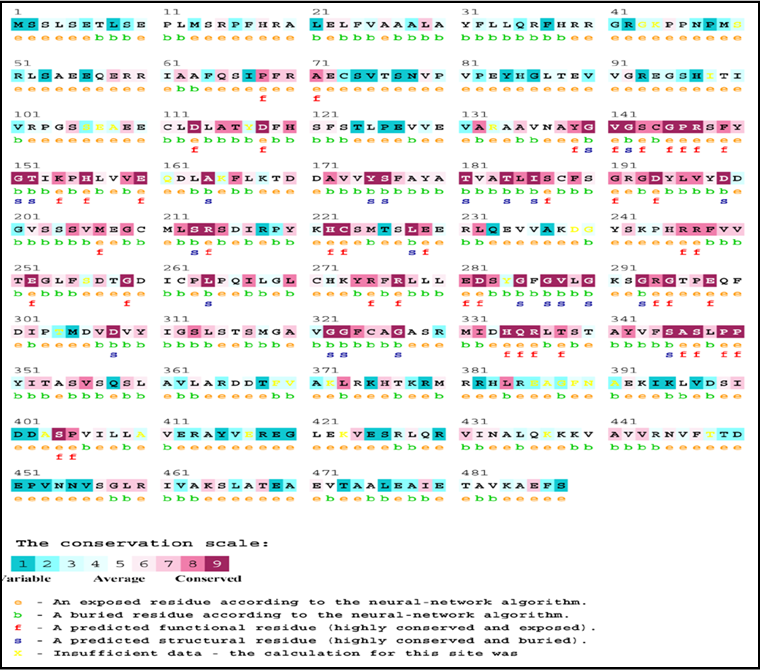

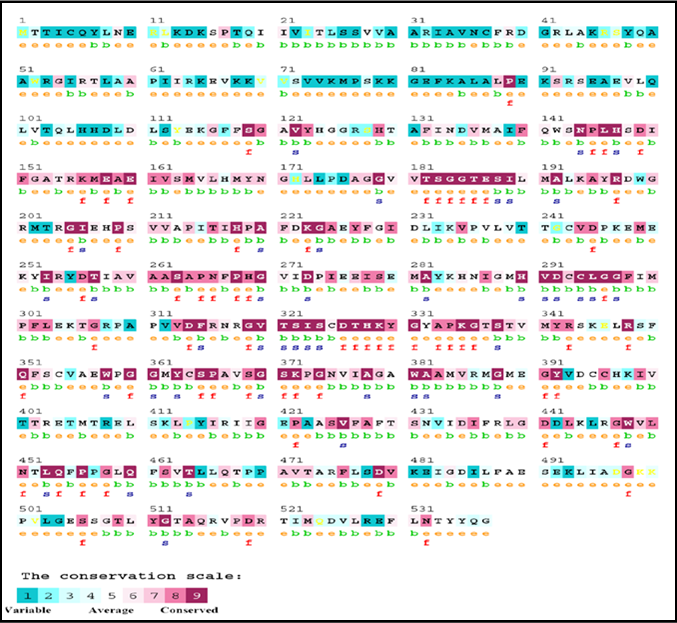
**

**(B)**

**
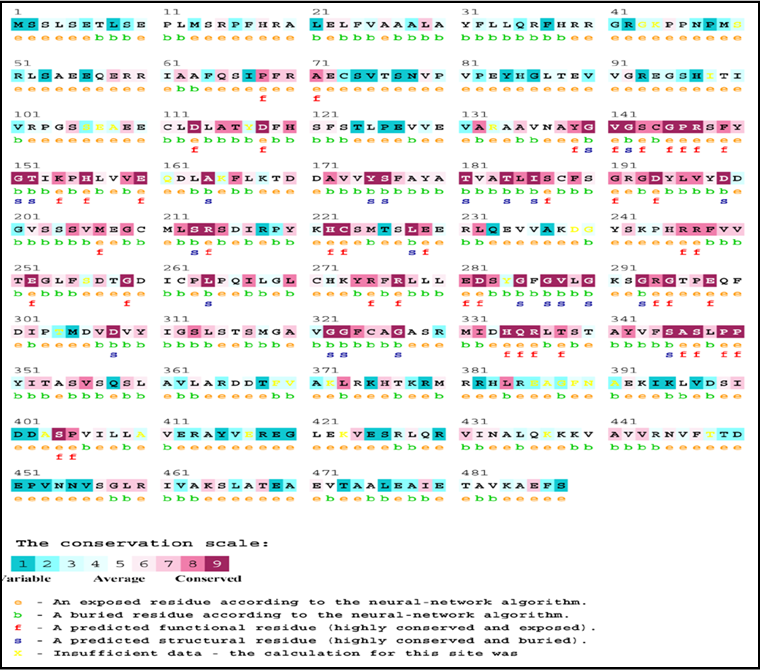

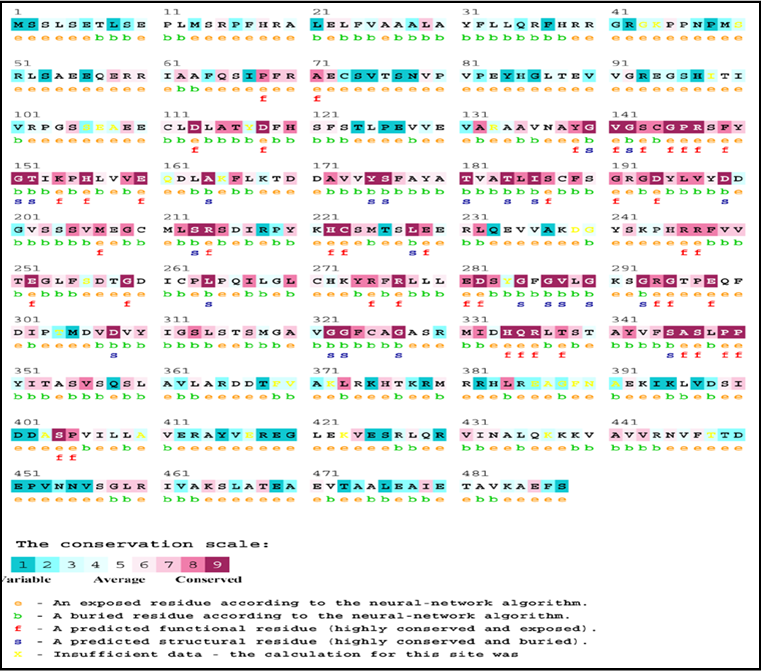
**

**(C)**

**
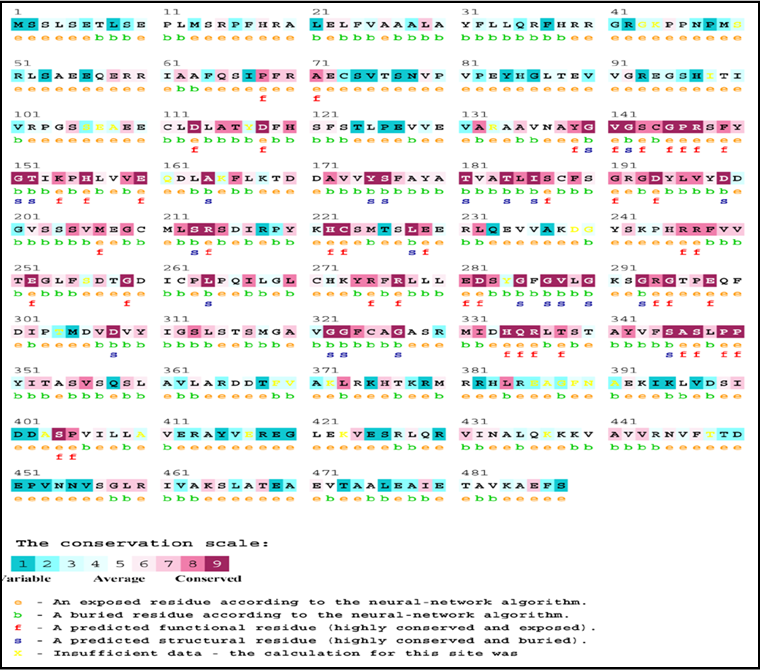

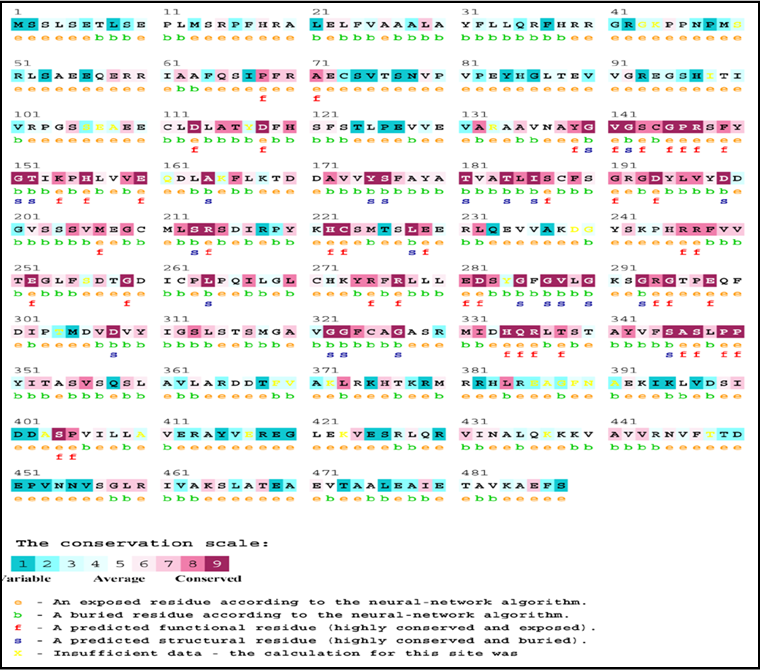
**

**(D)**

**Fig S1 A-D) CONSURF analysis showing the conservedness of IPCS, SPL, SPT1 and SPT2 proteins belonging to *L.major* (Amino-acids with dark pink color are highly conserved while one’s colored blue are least conserved)**

| **Codon** | **Count** | **RSCU** | **Codon** | **Count** | **RSCU** | **Codon** | **Count** | **RSCU** | **Codon** | **Count** | **RSCU** |
| --- | --- | --- | --- | --- | --- | --- | --- | --- | --- | --- | --- |
| UUU(F) | 2 | 0.4 | UCU(S) | 8 | 1.23 | UAU(Y) | 3 | 1 | UGU(C) | 5 | 0.77 |
| UUC(F) | 8 | 1.6 | UCC(S) | 11 | 1.69 | UAC(Y) | 3 | 1 | UGC(C) | 8 | 1.23 |
| UUA(L) | 4 | 1.33 | UCA(S) | 8 | 1.23 | UAA(*) | 2 | 1.33 | UGA(*) | 10 | 0.91 |
| UUG(L) | 8 | 2.67 | UCG(S) | 8 | 1.23 | UAG(*) | 1 | 0.67 | UGG(W) | 12 | 1.09 |
| CUU(L) | 3 | 1 | CCU(P) | 5 | 1.18 | CAU(H) | 3 | 0.67 | CGU(R) | 14 | 1.83 |
| CUC(L) | 1 | 0.33 | CCC(P) | 4 | 0.94 | CAC(H) | 6 | 1.33 | CGC(R) | 7 | 0.91 |
| CUA(L) | 0 | 0 | CCA(P) | 2 | 0.47 | CAA(Q) | 5 | 1.67 | CGA(R) | 11 | 1.43 |
| CUG(L) | 2 | 0.67 | CCG(P) | 6 | 1.41 | CAG(Q) | 1 | 0.33 | CGG(R) | 8 | 1.04 |
| AUU(I) | 2 | 0.86 | ACU(T) | 10 | 1.33 | AAU(N) | 2 | 2 | AGU(S) | 2 | 0.31 |
| AUC(I) | 4 | 1.71 | ACC(T) | 6 | 0.8 | AAC(N) | 0 | 0 | AGC(S) | 2 | 0.31 |
| AUA(I) | 1 | 0.43 | ACA(T) | 8 | 1.07 | AAA(K) | 4 | 1.14 | AGA(R) | 4 | 0.52 |
| AUG(M) | 5 | 1 | ACG(T) | 6 | 0.8 | AAG(K) | 3 | 0.86 | AGG(R) | 2 | 0.26 |
| GUU(V) | 4 | 1 | GCU(A) | 11 | 1.47 | GAU(D) | 6 | 1.2 | GGU(G) | 7 | 1.04 |
| GUC(V) | 3 | 0.75 | GCC(A) | 5 | 0.67 | GAC(D) | 4 | 0.8 | GGC(G) | 9 | 1.33 |
| GUA(V) | 1 | 0.25 | GCA(A) | 3 | 0.4 | GAA(E) | 4 | 1.6 | GGA(G) | 5 | 0.74 |
| GUG(V) | 8 | 2 | GCG(A) | 11 | 1.47 | GAG(E) | 1 | 0.4 | GGG(G) | 6 | 0.89 |

**(A)**

| **Codon** | **Count** | **RSCU** | **Codon** | **Count** | **RSCU** | **Codon** | **Count** | **RSCU** | **Codon** | **Count** | **RSCU** |
| --- | --- | --- | --- | --- | --- | --- | --- | --- | --- | --- | --- |
| UUU(F) | 19.1 | 1.27 | UCU(S) | 7.6 | 1.01 | UAU(Y) | 10.1 | 1.19 | UGU(C) | 15.4 | 1.21 |
| UUC(F) | 11 | 0.73 | UCC(S) | 8.6 | 1.15 | UAC(Y) | 6.9 | 0.81 | UGC(C) | 10 | 0.79 |
| UUA(L) | 8.9 | 0.79 | UCA(S) | 11.6 | 1.55 | UAA(*) | 6.3 | 0.96 | UGA(*) | 10.7 | 1.63 |
| UUG(L) | 9 | 0.8 | UCG(S) | 3.9 | 0.52 | UAG(*) | 2.7 | 0.41 | UGG(W) | 15.9 | 1 |
| CUU(L) | 18.4 | 1.64 | CCU(P) | 11 | 1.24 | CAU(H) | 18.3 | 1.13 | CGU(R) | 5 | 0.75 |
| CUC(L) | 9.9 | 0.88 | CCC(P) | 11.1 | 1.26 | CAC(H) | 14 | 0.87 | CGC(R) | 2.7 | 0.41 |
| CUA(L) | 5.9 | 0.52 | CCA(P) | 10.4 | 1.18 | CAA(Q) | 19 | 1.32 | CGA(R) | 4.3 | 0.64 |
| CUG(L) | 15.4 | 1.37 | CCG(P) | 2.9 | 0.32 | CAG(Q) | 9.9 | 0.68 | CGG(R) | 6.1 | 0.92 |
| AUU(I) | 9.9 | 1.34 | ACU(T) | 8.7 | 1.26 | AAU(N) | 11.3 | 1.14 | AGU(S) | 7.7 | 1.03 |
| AUC(I) | 6.6 | 0.9 | ACC(T) | 6.6 | 0.95 | AAC(N) | 8.4 | 0.86 | AGC(S) | 5.6 | 0.75 |
| AUA(I) | 5.6 | 0.76 | ACA(T) | 8.3 | 1.2 | AAA(K) | 16.7 | 1.3 | AGA(R) | 14.3 | 2.14 |
| AUG(M) | 6.3 | 1 | ACG(T) | 4.1 | 0.6 | AAG(K) | 9 | 0.7 | AGG(R) | 7.6 | 1.14 |
| GUU(V) | 10.7 | 1.06 | GCU(A) | 14.1 | 1.72 | GAU(D) | 10 | 1.17 | GGU(G) | 8.9 | 1.11 |
| GUC(V) | 5.6 | 0.55 | GCC(A) | 8.6 | 1.04 | GAC(D) | 7.1 | 0.83 | GGC(G) | 7.4 | 0.93 |
| GUA(V) | 10 | 0.99 | GCA(A) | 7.6 | 0.92 | GAA(E) | 13.6 | 1.31 | GGA(G) | 9.1 | 1.14 |
| GUG(V) | 14.3 | 1.41 | GCG(A) | 2.6 | 0.31 | GAG(E) | 7.1 | 0.69 | GGG(G) | 6.6 | 0.82 |

**(B)**

| **Codon** | **Count** | **RSCU** | **Codon** | **Count** | **RSCU** | **Codon** | **Count** | **RSCU** | **Codon** | **Count** | **RSCU** |
| --- | --- | --- | --- | --- | --- | --- | --- | --- | --- | --- | --- |
| UUU(F) | 10.3 | 0.74 | UCU(S) | 3 | 0.39 | UAU(Y) | 14 | 1.44 | UGU(C) | 9.3 | 1.4 |
| UUC(F) | 17.5 | 1.26 | UCC(S) | 8.8 | 1.14 | UAC(Y) | 5.5 | 0.56 | UGC(C) | 4 | 0.6 |
| UUA(L) | 2.8 | 0.34 | UCA(S) | 12.3 | 1.6 | UAA(*) | 4 | 1.41 | UGA(*) | 3.5 | 1.24 |
| UUG(L) | 12 | 1.48 | UCG(S) | 4 | 0.52 | UAG(*) | 1 | 0.35 | UGG(W) | 14.3 | 1 |
| CUU(L) | 5.3 | 0.65 | CCU(P) | 6 | 0.82 | CAU(H) | 7 | 0.9 | CGU(R) | 1.5 | 0.27 |
| CUC(L) | 6.3 | 0.77 | CCC(P) | 7 | 0.96 | CAC(H) | 8.5 | 1.1 | CGC(R) | 1.3 | 0.22 |
| CUA(L) | 5 | 0.62 | CCA(P) | 15.5 | 2.12 | CAA(Q) | 11 | 0.85 | CGA(R) | 2.5 | 0.45 |
| CUG(L) | 17.5 | 2.15 | CCG(P) | 0.8 | 0.1 | CAG(Q) | 14.8 | 1.15 | CGG(R) | 6 | 1.07 |
| AUU(I) | 8.5 | 1.12 | ACU(T) | 6 | 1 | AAU(N) | 6 | 0.75 | AGU(S) | 9 | 1.17 |
| AUC(I) | 10.3 | 1.35 | ACC(T) | 10.3 | 1.71 | AAC(N) | 6 | 1.25 | AGC(S) | 9 | 1.17 |
| AUA(I) | 4 | 0.53 | ACA(T) | 5 | 0.83 | AAA(K) | 18.5 | 1.18 | AGA(R) | 12.3 | 2.19 |
| AUG(M) | 17.5 | 1 | ACG(T) | 2.8 | 0.46 | AAG(K) | 12.8 | 0.82 | AGG(R) | 10 | 1.79 |
| GUU(V) | 6.5 | 0.81 | GCU(A) | 12.8 | 1.44 | GAU(D) | 15.8 | 1.31 | GGU(G) | 6 | 0.64 |
| GUC(V) | 8.8 | 1.09 | GCC(A) | 11.3 | 1.27 | GAC(D) | 8.3 | 0.69 | GGC(G) | 11 | 1.18 |
| GUA(V) | 5.3 | 0.65 | GCA(A) | 10.5 | 1.18 | GAA(E) | 9 | 1 | GGA(G) | 13.5 | 1.45 |
| GUG(V) | 11.8 | 1.46 | GCG(A) | 1 | 0.11 | GAG(E) | 9 | 1 | GGG(G) | 6.8 | 0.72 |

**(C)**

**Table S4A-C:** **Relative synonymous codon usage for IPCS, SPL and SPT genes**
